# Supplementary material for: The phosphate exporter XPR1 promotes gasdermin D–independent mature IL-1β secretion
Source: J Biol Chem. 2026 Sep 12;302(10):113547. doi: 10.1016/j.jbc.2026.113547 (PMC13676985; doi:10.1016/j.jbc.2026.113547)
Supplement: Supplementary file 1 [file mmc3.docx]

**Fig. S1. Related to Figure 1, Transcriptomic analysis of THP-1 cells following differentiation with PMA.**

THP-1 cells were treated or not with PMA (300 ng/mL) for 3 h, washed, and further differentiated for 18 h prior to RNAseq analysis. Gene expression levels (read counts) in PMA-treated cells are indicated on the right.

**Fig. S2. Related to Figure 1, Pro-IL-1β processing in THP-1 stimulated with LPS alone is dependent on both caspase 1 and NLRP3, and independent of caspase 8, 4 or 5.**

PMA-differentiated THP-1 cells (WT or *CASP1*, *NLRP3*, *CASP8*, *CASP4* or *CASP5* KO) were stimulated with LPS (10 ng/mL) or not for 24 h; nigericin (10µM) was added 4 h after LPS. At the end of the stimulation, supernatants were collected, and cells were resuspended in 200 µL of medium and lysed by single freeze-thaw cycle. Samples for immunoblotting were prepared using Laemmli Buffer (4x) containing β-mercaptoethanol, denaturated for 10 min at 95°C, separated by Bis-Tris denaturing SDS-PAGE and transferred onto nitrocellulose membranes.

Top panel. Detection of proIL-1β and mature IL-1β in cell lysates and corresponding culture supernatants. Membranes were probed with the indicated antibodies. Immunoblots were intentionally overexposed to demonstrate the absence of mature IL-1β in *CASP1*^-/-^ and *NLRP3*^-/-^ THP-1 cells. Because the KO cell lines were generated by single-cell cloning, some clonal variability was observed in the basal levels of pro-IL-1β and mature IL-1β in PMA-treated, unstimulated cells. Importantly, this variability was clone-dependent rather than genotype-dependent. As shown in Figure S1, PMA-differentiation upregulated pro-IL-1β expression.

Bottom panel. Concentrations (ng/mL) of mature IL-1β measured in the supernatants of the corresponding samples by ELISA. Data are presented as mean ± SD of technical triplicates.

The experiment shown is representative of at least three independent experiments.

**Fig. S3. Related to Figure 1,2,3 and 4. Immunoblot validation of gene inactivation confirmed by targeted sequencing.**

Different KO clones were validated by immunoblot analysis.

(A) Gene inactivation in the *CASP1-*, *NLRP3-*, *CASP8-*, *CASP4*-KO clones analyzed in Figure S2 was confirmed by immunoblotting. Reliable immunoblot-based validation could not be established for caspase-5; however, gene inactivation was confirmed by targeted sequencing (Table S1).

(B) Gene inactivation in the *GSDMD*-KO clone used in Figures 1- 4 was confirmed by immunoblotting. Clone 1 (indicated in bold) is the clone used throughout the manuscript.

(C) GSDMD gene inactivation was confirmed by immunoblotting in the clones presented in Figures 3 and 4. Clones indicated in bold were used in the manuscript. Reliable immunoblot-based validation could not be established for XPR1 and KIDINS220; however, gene inactivation was confirmed by targeted sequencing (Table S1).

**Fig. S4. Related to Figure 2. Characterization of WT and *GSDMD*^-/-^ THP-1 cells**

**(A) Caspase-1 cleavage.** PMA-differentiated WT and *GSDMD*^-/-^THP-1 cells were stimulated with LPS (10 ng/mL) for 4 h30 or left unstimulated. Nigericin (10 µM) was added 4 h after LPS as a positive control for inflammasome activation. Cells were lysed directly in Laemmli buffer containing β-mercaptoethanol, denaturated for 10 min at 95°C, and analyzed by denaturing Bis-Tris SDS-PAGE followed by transfer to nitrocellulose membranes. Immunoblotting was performed to detect β-actin, pro-caspase-1 and cleaved caspase-1 in cell lysates. The experiment shown is representative of two independent experiments.

**(B) Caspase-1 activity.** PMA-differentiated WT and *GSDMD*^-/-^THP-1 cells were stimulated with LPS (10 ng/mL) for 5 h or left unstimulated. Nigericin (10 µM) was added 4 h after LPS as a positive control for inflammasome activation. Caspase-1 activity was measured in cells and corresponding culture supernatants using the Caspase-Glo 1 Inflammasome Assay (Promega). Data are presented as mean ± SD of technical triplicates. The experiment shown is representative of at least three independent experiments.

**(C) Immunoblot validation of the results presented in Figure 2.**

Culture supernatants and cell lysates were collected using the same experimental procedure described in Figure 2. Samples were mixed with 4x-Laemmli buffer containing β-mercaptoethanol, denaturated for 10 min at 95°C, and analyzed by denaturing Bis-Tris SDS-PAGE followed by transfer to nitrocellulose membranes. Immunoblotting was performed to detect β-actin, pro-IL-1β and mature IL-1β in cell lysates and corresponding culture supernatants. The experiment shown is representative of two independent experiments.

Fig. S5. Validation that the human IL-1β Cyto-Set ELISA kit detects human mature IL-1β but not pro-IL-1β.

HEK293T cells were transfected with plasmids encoding either human mature IL-1β or pro-IL-1β. After 3 days, cells were either collected in the Laemmli buffer (reducing conditions) for Western blot analysis or lysed by a single freeze-thaw cycle. These thawed cell lysates were centrifuged and the supernatant collected for ELISA.

Top panels. Detection of pro-IL-1β and mature IL-1β in transfected HEK293T cells by immunoblotting. A single membrane was cut into three pieces and probed with the indicated antibodies.

Bottom panel. Concentrations (pg/mL) of IL-1β measured in cell lysates with three different ELISA kits.

The Quantikine pro-IL-1β kit detected pro-IL-1β but not mature IL-1β. The Ready-SET-Go! kit detected both mature IL-1β and pro-IL-1β. The Human IL-1β Cyto-Set kit detected mature IL-1β and produced only a very weak signal in lysates from HEK293T cells expressing pro-IL-1β. We cannot exclude the possibility that this signal reflects a low level of pro-IL-1β processing in HEK293T cells. The Human IL-1β Cyto-Set kit was used throughout this study.

Fig. S6. Related to Figure 3, Mature IL-1β–enriched cell sorting and quality control of the CRISPR/Cas9 screening.

(A) Results of cell sorting performed during the CRISPR/Cas9 screening. For visualization purposes, replicates 1, 2, and 3 were downsampled to 30,000 cells (total number of analyzed cells: Rep1 – 21,764,670; Rep2 – 19,624,963; Rep3 – 19,918,967).

(B) Quality control analysis using MAGeCK. Percentages of reads mapped to the Brunello library were >65% (consistent with standard for this type of screen). Library representation (defined as the proportion of different gRNAs from the library detected among the mapped reads) was close to 100% in control cell populations.

**Fig. S7. Related to Figure 4, XPR1 positively regulates GSDMD-independent mature IL-1β secretion efficiency.**

(A) ELISA kinetics from the independent experiments used to calculate secretion efficiency at 24 h after LPS stimulation in Figure 4A. Data are presented as mean ± SD of technical triplicates.

(B) ELISA kinetics from the independent experiments used to calculate secretion efficiency at 24 h after LPS stimulation in Figure 4A. Data are presented as mean ± SD of technical triplicates.

Fig. S8. Related to Figure 4, XPR1 re-expression restores mIL-1β secretion in *GSDMD*^-/-^ *XPR1*^-/-^ THP-1 cells.

GSDMD^-/-^XPR1^-/-^ cells were transduced with lentiviral vectors expressing human XPR1 at increasing dilutions.

(A) **XPR1 expression following lentiviral transduction**. Representative immunofluorescence images of *GSDMD*^-/-^ and *GSDMD*^-/-^ *XPR1*^-/-^ cells either non-transduced or transduced with XPR1 lentiviral vectors. Following PMA treatment, cells were stained with an anti-XPR1 (green) and DAPI (blue). Images were acquired using identical exposure settings to allow direct comparison. Magnification 20x; scale bar, 10 µm.

XPR1 transduction resulted in marked overexpression compared with endogenous XPR1 expression in *GSDMD*^-/-^ cells. Serial dilution of the lentiviral vectors reduced the proportion of XPR1-positive cells rather than the level of XPR1 expression in individual transduced cells. Despite testing several promoters, physiological XPR1 expression could not be achieved following lentiviral transduction.

(B) **Effect or XPR1 re-expression on mature IL-1β secretion**. Time course of mIL-1β secretion measured by ELISA in cell lysates and culture supernatants following LPS stimulation of *GSDMD*^-/-^and *GSDMD*^-/-^ *XPR1*^-/-^ cells either non-transduced or transduced with XPR1 lentiviral vectors. Data are presented as mean ± SD of technical triplicates. Transduction with undiluted XPR1 lentivirus (1:1) restored mIL-1β secretion, reaching a secretion efficiency of 61% at 24 hours after LPS stimulation, comparable to that observed in *GSDMD*^-/-^ cells. Progressive dilution of the lentiviral vectors reduced the proportion of XPR1-expressing cells and was associated with a corresponding decrease in mIL-1β secretion efficiency.

Fig. S9. Validation that the antibody used for the CRISPR library screening specifically recognizes human mature IL-1β but not pro-IL-1β.

HEK293T cells were transfected with plasmids encoding either human mature IL-1β or pro-IL-1β. After 3 days, cells were collected, fixed, permeabilized, and stained with a rabbit IgG Isotype Control (Alexa Fluor 488 (AF488) conjugate) as negative control (Isoctrl), a rabbit anti-cleaved IL-1β antibody conjugated to Alexa Fluor 488 (AF488) or a mouse FITC-conjugated anti-human IL-1β antibody recognizing both pro- and mature IL-1β. The AF488-conjugated anti-cleaved IL-1β specifically detected mature IL-1β into cells.

Table S1. Genetic validation by targeted sequencing of all gene-invalidated THP-1 cell clones used in this study.

For each clone, the WT reference sequence of the targeted gene is shown on top with the sgRNA target site indicated in red. Sequences of the edited alleles are shown below; deletions are represented by dots, and insertions are indicated in a larger font.

(1) Clone used for data shown in Figs. 1–4.

(2) Clone used for data shown in Figs. 3 and 4.

(3) Clone used for data shown in Fig. 4.

(4-8) Clones used for data shown in Fig. S2.

| **THP-1 clone** | **Gene** | **Sequence** | | **Length of encoded protein (AA)** |
| --- | --- | --- | --- | --- |
| ***GSDMD*^-/-^ cl1 ^(1)^** | *GSDMD* | WT | ATGGGGTCGGCCTTTGAGCGGGTAGTCCGGAGAGTGGTCCAGGAGCTGGACCAT | 484 |
|  |  | Allele 1 | ATGGGGTCGGCCTTTG**A**AGCGGGTAGTCCGGAGAGTGGTCCAGGAGCTGGACCAT | 41 |
|  |  | Allele 2 | ATGGGGTCGGCCTTTGA**..**GGGTAGTCCGGAGAGTGGTCCAGGAGCTGGACCAT | 40 |
| *GSDMD*^-/-^ cl2 | *GSDMD* | WT | CAGGAGCTGGACCATGGTGGGGAGTTCATCCCTGTGACCAGCCTGCAGAGCTCCACT | 484 |
|  |  | Allele 1 | CAGGAGCTGGACCATGG**.**GGGGAGTTCATCCCTGTGACCAGCCTGCAGAGCTCCACT | 24 |
|  |  | Allele 2 | CAGGAGCTGGACCATG**GT**GTGGGGAGTTCATCCCTGTGACCAGCCTGCAGAGCTCCACT | 25 |
| GSDMD^-/-^ XPR1^-/-^ cl1 | *XPR1* | WT | TACTAGATTGCTGGATTCCTCGGGATATTGTGGTGCCTGAGCCTTCTGGCATGCTTCTTT | 696 |
|  |  | Allele 1 | TACTAGATTGCTGGATTCC**........**TTGTGGTGCCTGAGCCTTCTGGCATGCTTCTTT | 335 |
|  |  | Allele 2 | TACTAGATTGCTGGATTCCTCGG**..**TATTGTGGTGCCTGAGCCTTCTGGCATGCTTCTTT | 337 |
|  |  | Allele 3 | TACTAGATTGCTGGATTCCTCGGG**.**TATTGTGGTGCCTGAGCCTTCTGGCATGCTTCTTT | 327 |
|  | *GSDMD* | WT | ATGGGGTCGGCCTTTGAGCGGGTAGTCCGGAGAGTGGTCCAGGAGCTGGACCAT | 484 |
|  |  | Allele 1 | ATGGGGTCGGCCTTT**.**AGCGGGTAGTCCGGAGAGTGGTCCAGGAGCTGGACCAT | 7 |
|  |  | Allele 2 | ATGGGGTCGGCCTTTGA**A**GCGGGTAGTCCGGAGAGTGGTCCAGGAGCTGGACCAT | 41 |
|  |  | Allele 3 | ATGGGGTCGGCCTTT**.....**GGTAGTCCGGAGAGTGGTCCAGGAGCTGGACCAT | 39 |
| **GSDMD^-/-^ XPR1^-/-^ cl2 ^(2)^** | *XPR1* | WT | GAAGTTCGCCGAGCACCTCTCCGCGCACATCACTCCCGAGTGGAGGAAGCAATACATCCA | 696 |
|  |  | Allele 1 | GAAGTTCGCCGAGCACCTCTCCGCGC**....................**AAGCAATACATCCA | 15 |
|  |  | Allele 2 | GAAGTTCGCCGAGCACCTCTCCGCGCACATCACT**.**CCGAGTGGAGGAAGCAATACATCCA | 46 |
|  | *GSDMD* | WT | ATGGGGTCGGCCTTTGAGCGGGTAGTCCGGAGAGTGGTCCAGGAGCTGGACCAT | 484 |
|  |  | Allele 1 | ATGGGGTCGGCCTTT**..**GCGGGTAGTCCGGAGAGTGGTCCAGGAGCTGGACCAT | 40 |
|  |  | Allele 2 | ATGGGGTCGGCCTTTG**.**GCGGGTAGTCCGGAGAGTGGTCCAGGAGCTGGACCAT | 7 |
|  |  | Allele 3 | ATGGGGTCGGCCTTTGA**..**GGGTAGTCCGGAGAGTGGTCCAGGAGCTGGACCAT | 40 |
| **GSDMD^-/-^ KIDINS220^-/-^ cl1 ^(3)^** | *KIDINS220* | WT | TGTAGTGGGATTGGCCTTTGTGTTGAACTGTCGTACATGGTGGCAAGTGCTGGACTCGCT | 1771 |
|  |  | Allele 1 | TGTAGTGGGATTGGCCTTTGTGTTGAACTGTCGTAC**.**TGGTGGCAAGTGCTGGACTCGCT | 718 |
|  |  | Allele 2 | TGTAGTGGGATTGGCCTTTGTGTTGAACTGTCGTACA**.**GGTGGCAAGTGCTGGACTCGCT | 718 |
|  | *GSDMD* | WT | ATGGGGTCGGCCTTTGAGCGGGTAGTCCGGAGAGTGGTCCAGGAGCTGGACCAT | 484 |
|  |  | Allele 1 | ATGGGGTCGGCCTTTGA**.**CGGGTAGTCCGGAGAGTGGTCCAGGAGCTGGACCAT | 7 |
|  |  | Allele 2 | ATG**................**GGGTAGTCCGGAGAGTGGTCCAGGAGCTGGACCAT | 2 |
|  |  | Allele 3 | ATGGGGTCGGCCTTTG**A**AGCGGGTAGTCCGGAGAGTGGTCCAGGAGCTGGACCAT | 41 |
| GSDMD^-/-^ KIDINS220^-/-^ cl2 | *KIDINS220* | WT | GTGCACATCGTAGAGGAACTACTGAAATGTGGGGTTAACTTGGAGCACCGTGATATGGGAGGA | 1771 |
|  |  | Allele 1 | GTGCACATCGTAGAGGAACTACTGAAATGTGG**.....**ACTTGGAGCACCGTGATATGGGAGGA | 98 |
|  |  | Allele 2 | GTGCACATCGTAGAGGAACTACTGAAATGTGGGGTT**A**AACTTGGAGCACCGTGATATGGGAGGA | 100 |
|  | *GSDMD* | WT | ATGGGGTCGGCCTTTGAGCGGGTAGTCCGGAGAGTGGTCCAGGAGCTGGACCAT | 484 |
|  |  | Allele 1 | ATGGGGTCGGCCTTTG**.**GCGGGTAGTCCGGAGAGTGGTCCAGGAGCTGGACCAT | 7 |
|  |  | Allele 2 | ATGGGGTCGGCCTTTGA**.....**TAGTCCGGAGAGTGGTCCAGGAGCTGGACCAT | 39 |
| **CASP1 ^-/-^ cl1 ^(4)^** | *CASP1* | WT | AGGTCCTGAAGGAGAAGAGAAAGCTGTTTATCCGTTCCATGGGTGAAGGTACAATAAATG | 404 |
|  |  | Allele 1 | AGGTCCTGAAGGAGAAGAGAAAGCTGTTTATCCGT**.......**GTGAAGGTACAATAAATG | 19 |
|  |  | Allele 2 | AGGTCCTGAAGGAGAAGAGAAAGCTGTTTATCC**.......**GGGTGAAGGTACAATAAATG | 19 |
| **CASP8 ^-/-^ cl1 ^(5)^** | *CASP8* | WT | GGCCTCCCTCAAGTTCCTGAGCCTGGACTACATTCCGCAAAGGAAGCAAGAACCCATCAA | 479 |
|  |  | Allele 1 | GGCCTCCCTCAAGTTCCTGAGCCTGGA**.......**CCGCAAAGGAAGCAAGAACCCATCAA | 40 |
|  |  | Allele 2 | GGCCTCCCTCAAGTTCCTGAGCCTGGACTA**.......**CAAAGGAAGCAAGAACCCATCAA | 40 |
|  |  | Allele 3 | GGCCTCCCTCAAGTTCCTGAGCCTGGACTACATTCCG**.**AAAGGAAGCAAGAACCCATCAA | 42 |
| **CASP4 ^-/-^ cl1 ^(6)^** | *CASP4* | WT | GGGTCATGGCAGACTCTATGCAAGAGAAGCAACGTATGGCAGGACAAATGCTTCTTCAAA | 377 |
|  |  | Allele 1 | GGGTCATGGCAGACTCTATGCAAGAGAAGCAACGT**.....**AGGACAAATGCTTCTTCAAA | 76 |
|  |  | Allele 2 | GGGTCATGGCAGACTCTATGCAAGAGAAGCAAC**.....**G**A**AGGACAAATGCTTCTTCAAA | 76 |
|  |  | Allele 3 | GGGTCATGGCAGACTC**........................................**CAAA | 66 |
| **CASP5 ^-/-^ cl1 ^(7)^** | *CASP5* | WT | AAAGATCACCAGTGTAAAACCTCTTCTGCAAATCGAGGCTGGACCACCTGAGTCAGCAGAATCTACAAATATACTCAAACTT | 434 |
|  |  | Allele 1 | AAAGATCACCAGTGTAAAACCTCTTCT**.....................................**ACAAATATACTCAAACTT | 484 |
| **NLRP3 ^-/-^ cl1 ^(8)^** | *NLRP3* | WT | AGACCATGTGGATCTAGCCACGCTAATGATCGACTTCAATGGGGAGGAGAAGGCGTGGGC | 1037 |
|  |  | Allele 1 | AGACCATGTGGATCTAGCC**.............**ACTTCAATGGGGAGGAGAAGGCGTGGGC | 101 |
|  |  | Allele 2 | AGACCATGT**...............................**GGGGAGGAGAAGGCGTGGGC | 95 |
|  |  | Allele 3 | AGACCATGTGGATCTAGCCACGC**T**TAATGATCGACTTCAATGGGGAGGAGAAGGCGTGGGC | 84 |

Table S2. Genetic validation of the GSDMD^-/-^ THP-1 clones knocked-in with XPR1 mutants.

For each clone, the WT reference sequences of the targeted genes are shown on top with the sgRNA target site indicated in red. For the *XPR1* L218S and KSC mutations, the HDR donor templates are shown, with the introduced mutation(s) in a larger font. For each of the four THP-1 clones shown, all sequencing reads spanning the targeted *XPR1* locus were identical and contained the desired mutation(s).

For each clone, the WT reference sequence of the targeted gene is shown on top with the sgRNA target site indicated in red. Sequences of the edited alleles are shown below; deletions are represented by dots, and insertions are indicated in a larger font.

(1) Clones used for data shown in Fig. 4.

| **THP-1 clone** | **Gene** | **Sequence** | | **Length of encoded protein (AA)** |
| --- | --- | --- | --- | --- |
| ***GSDMD^-/-^ XPR1^L218S/L218S^* cl1 *^(1)^*** | *XPR1* | WT | GAACTTGAAGATGGTGACAGACAAAAGGCTATGAAGCGTTTACGGGTCCCCCCTCTGGGAGCTGCTCAGGTTAGTATTTGGTTCCAGTAGTT | 696 |
|  |  | HDR donor template | GAACTTGAAGATGGTGACAGACAAAAGGCTATGAAGCGTT**C**ACGGGTCCCCCCTCTGGGAGCTGCTCAGGTTAGTATTTGGTTCCAGTAGTT | 696 |
|  | *GSDMD* | WT | ATGGGGTCGGCCTTTGAGCGGGTAGTCCGGAGAGTGGTCCAGGAGCTGGACCAT | 484 |
|  |  | Allele 1 | **..................**CGGGTAGTCCGGAGAGTGGTCCAGGAGCTGGACCAT | / |
|  |  | Allele 2 | ATGGGGTCGGCCTTTGA**A**GCGGGTAGTCCGGAGAGTGGTCCAGGAGCTGGACCAT | 40 |
| *GSDMD^-/-^ XPR1^L218S/L218S^* cl2 | *XPR1* | WT | GAACTTGAAGATGGTGACAGACAAAAGGCTATGAAGCGTTTACGGGTCCCCCCTCTGGGAGCTGCTCAGGTTAGTATTTGGTTCCAGTAGTT | 696 |
|  |  | HDR donor template | GAACTTGAAGATGGTGACAGACAAAAGGCTATGAAGCGTT**C**ACGGGTCCCCCCTCTGGGAGCTGCTCAGGTTAGTATTTGGTTCCAGTAGTT | 696 |
|  | *GSDMD* | WT | ATGGGGTCGGCCTTTGAGCGGGTAGTCCGGAGAGTGGTCCAGGAGCTGGACCAT | 484 |
|  |  | Allele 1 | ATGGGGTCGGCCTTTGA**.**CGGGTAGTCCGGAGAGTGGTCCAGGAGCTGGACCAT | 6 |
|  |  | Allele 2 | ATGGGGTCGGCCTTTGA**A**GCGGGTAGTCCGGAGAGTGGTCCAGGAGCTGGACCAT | 40 |
| ***GSDMD^-/-^ XPR1^KSC/KSC^* cl1 ^(1)^** | *XPR1* | WT | TCATTTCTTTCTGTAGAATCTGAATTTTACAGGGTTTCGAAAAATCCTGAAAAAGCATGACAAGATCCTGGAAACATCTCGTGGAGCAGATTGGCGAGTGGCTCACGTAGAG | 696 |
|  |  | HDR donor template | TCATTTCTTTCTGTAGAATCTGAATTTTACAGGGTTTCGA**GC**AATCCTG**GC**AAAGCATGAC**GC**GATCCTGGAAACATCTCGTGGAGCAGATTGGCGAGTGGCTCACGTAGAG | 696 |
|  | *GSDMD* | WT | ATGGGGTCGGCCTTTGAGCGGGTAGTCCGGAGAGTGGTCCAGGAGCTGGACCAT | 484 |
|  |  | Allele 1 | **..................**CGGGTAGTCCGGAGAGTGGTCCAGGAGCTGGACCAT | / |
|  |  | Allele 2 | ATGGGGTCGGCCTTTG**.....**GTAGTCCGGAGAGTGGTCCAGGAGCTGGACCAT | 38 |
|  |  | Allele 3 | ATGGGGTCGGCCTTT**T**AGCGGGTAGTCCGGAGAGTGGTCCAGGAGCTGGACCAT | 4 |
| *GSDMD^-/-^ XPR1^KSC/KSC^* cl2 | *XPR1* | WT | TCATTTCTTTCTGTAGAATCTGAATTTTACAGGGTTTCGAAAAATCCTGAAAAAGCATGACAAGATCCTGGAAACATCTCGTGGAGCAGATTGGCGAGTGGCTCACGTAGAG | 696 |
|  |  | HDR donor template | TCATTTCTTTCTGTAGAATCTGAATTTTACAGGGTTTCGA**GC**AATCCTG**GC**AAAGCATGAC**GC**GATCCTGGAAACATCTCGTGGAGCAGATTGGCGAGTGGCTCACGTAGAG | 696 |
|  | *GSDMD* | WT | ATGGGGTCGGCCTTTGAGCGGGTAGTCCGGAGAGTGGTCCAGGAGCTGGACCAT | 484 |
|  |  | Allele 1 | ATGGGGTCGGCCTTTG**..**CGGGTAGTCCGGAGAGTGGTCCAGGAGCTGGACCAT | 39 |
|  |  | Allele 2 | ATGGGGTCGGCCTTTGA**.**CGGGTAGTCCGGAGAGTGGTCCAGGAGCTGGACCAT | 6 |

**Table S3. KEY RESOURCES TABLE.**

| REAGENT or RESOURCE | SOURCE | IDENTIFIER |
| --- | --- | --- |
| Antibodies | | |
| Rabbit monoclonal anti-cleaved-IL-1β-AF488 (clone D3A3Z) | This paper | N/A |
| Mouse monoclonal anti-IL-1β-FITC (clone JK1B-1) | BioLegend | Cat #508206; RRID:AB_345362 |
| PE-conjugated anti-mouse IgG1 | Biolegend | Cat #405307; RRID:AB_315010 |
| Mouse monoclonal anti-vinculin | Sigma-Aldrich | Cat# V9131, RRID:AB_477629 |
| Rabbit monoclonal anti-β-Actin | Cell Signaling Technology | Cat#8457, RRID:AB_10950489 |
| Rabbit monoclonal anti- IL-1β | Cell Signaling Technology | Cat# 12703, RRID:AB_2737350 |
| Rabbit monoclonal anti-cleaved-IL-1β (clone D3A3Z) | Cell Signaling Technology | Cat# 83186, RRID:AB_2800010 |
| Rabbit monoclonal anti-GSDMD (clone E9S1X) | Cell Signaling Technology | Cat# 39754, RRID:AB_2916333 |
| Rabbit monoclonal anti-Caspase-1 (clone D7F10) | Cell Signaling Technology | Cat# 3866, RRID:AB_2069051 |
| Rabbit monoclonal anti-Cleaved Caspase-1 (Asp297) (clone D57A2) | Cell Signaling Technology | Cat#4199,  RRID |
| Rabbit monoclonal anti-Caspase-8 (clone D35G2) | Cell Signaling Technology | Cat# 4790, RRID:AB_10545768 |
| Rabbit anti-Caspase-4 | Cell Signaling Technology | Cat# 4450, RRID:AB_1950386 |
| Mouse monoclonal anti-NLRP3 | AdipoGen | Cat#AG-20B-0014,  RRID: AB_2490202 |
| Rabbit polyclonal anti-XPR1 | Sigma-Aldrich | Cat # HPA016557 |
| Chemicals, peptides, and recombinant proteins | | |
| Ultrapure LPS from *E. coli*, serotype 055:B5 | InvivoGen | Cat #tlrl-pb5lps |
| Nigericin | InvivoGen | Cat #tlrl-nig |
| PMA-Phorbol myristate acetate | InvivoGen | Cat #tlrl-pma |
| XRBD | Giovannini et al. ^27^ | N/A |
| TrypLE^TM^ Express Enzyme | ThermoFischer Scientific | Cat #1260510 |
| TransIT-LT1 | Mirus | MIR-2304 |
| TrueCut™ Cas9 Protein v2 | ThermoFischer Scientific | Cat #A36499 |
| Proteinase K from *Tritirachium album* | Merck | Cat #P2308 |
| KAPA HiFi Polymerase | Roche | Cat #KK2502 |
| AMPure XP Reagent | Beckman Coulter | Cat #A63881 |
| Alt-R Cas9 Electroporation Enhancer | IDT | Cat #1075915 |
| 4% paraformaldehyde/PBS | Invitrogen | Cat #FB002 |
| Critical commercial assays | | |
| BD Cytofix/Cytoperm Fixation/Permeabilization Kit | BD | Cat #554714 |
| QIAamp DNA Micro Kit | Qiagen | Cat #56304 |
| SG Cell Line 96-well Nucleofector^®^ Kit | Lonza | Cat #V4SC-3096 |
| Nextera XT Index Kit v2 Set A | Illumina | Cat # FC-131-2001 |
| MiSeq Reagent Kit v2 (500-cycles) | Illumina | Cat # MS-102-2003 |
| P3 Primary Cell 4D-Nucleofector™ X Kit S | Lonza | Cat # V4XP-3032 |
| IL-1β Human Matched Antibody Pair ELISA kit | ThermoFischer Scientific | Cat #CHC1213 |
| Human pro-IL-1 beta/IL-1F2 quantikine ELISA Kit | bio-techne | Cat #DLB00 |
| Human IL-1 beta Ready-SET-Go! Kit | ThermoFischer Scientific | Cat #88-7261-88 |
| IL1RA Human Matched Antibody Pair | ThermoFischer Scientific | Cat #CHC1183 |
| LDH-Glo Cytotoxicity Assay | Promega | Cat #J2381 |
| CellTox Green reagent | Promega | Cat #G8731 |
| CellTiter 96 aqueous non-radioactive cell proliferation assay | Promega | Cat #G5421 |
| Deposited data | | |
| RNASeq data | This paper | GSE117162 |
| Dataset from CRISPR-Cas9 screening (Fig. 3) | This paper | DOI : 10.17632/3nj4b7xpjw.1 |
| Experimental models: Cell lines | | |
| THP-1 cells | ATCC | TIB-202 |
| HEK-293 cells | ATCC | CRL-1573 |
| Experimental models: Organisms/strains | | |
| Oligonucleotides | | |
| Alt-R® CRISPR-Cas9 tracrRNA | IDT | Cat #1072534 |
| Recombinant DNA | | |
| Human Brunello CRISPR knockout pooled library | Addgene | Cat #73179; RRID:Addgene_73179 |
| psPAX2 | Addgene | Cat #12260; RRID:Addgene_12260 |
| pCMV-VSV-G | Addgene | Cat #8454; RRID:Addgene_8454 |
| Software and algorithms | | |
| Model-based Analysis of Genome-wide CRISPR-Cas9 Knockout (MAGeCK) | Li et al. ^52^ | <https://github.com/liulab-dfci/MAGeCK> |
| Other | | |
| Vivaspin 20 centrifugal concentrators | Sigma-Aldrich | Cat #Z614637 |
| 18-well chamber slide | Ibidi | Cat #81816 |

Dataset S1. sgRNA summary generated after MAGeCK-RRA analysis

Table generated by MAGeCK-RRA paired analysis providing detailed information for each individual sgRNA, including gene mapping, enrichment in sorted population (treatment) compared with the total population (control), and associated p-values.

Dataset S2. Gene summary generated after MAGeCK-RRA analysis

Table generated by MAGeCK-RRA paired analysis providing a ranking of all genes based on RRA scores, including positive and negative selection scores, p-values and False Discovery Rates (FDR).
